# Combined effects of increased water temperature and cyanobacterial compounds exert heterogeneous effects on survival and ecological processes in key freshwater species

**DOI:** 10.1007/s00442-022-05277-7

**Published:** 2022-11-07

**Authors:** Oloyede A. Adekolurejo, Matthew Floyd, Alison M. Dunn, Paul Kay, Andrew P. Dean, Christopher Hassall

**Affiliations:** 1 School of Biology, Faculty of Biological Sciences, University of Leeds, Leeds, LS2 9JT UK; 2 School of Geography, Faculty of Environment, University of Leeds, Leeds, LS2 9JT UK; 3 Department of Natural Sciences, Faculty of Science and Engineering, Manchester Metropolitan University, Manchester, M1 5GD UK

**Keywords:** Algal bloom, Toxin, Microcystin, Feeding, Survival, Freshwater

## Abstract

Climate change is increasing water temperature and intensifying the incidence of cyanobacterial blooms worldwide. However, the combined effects of increased temperature and microcystin concentrations as co-stressors on survival and ecological processes in freshwater species are unclear. Here, using purified MC-LR and crude extract of toxigenic *Microcystis aeruginosa*, we tested the individual and combined effects of three water temperatures (15, 20, 25 °C) and a range of environmentally relevant concentrations of dissolved microcystin and crude extract (0.01–10 µg·L^−1^) on survival, growth inhibition, grazing and predation rates in three freshwater species: phytoplankton (*Scenedesmus quadricauda*), zooplankton (*Daphnia pulex*), and an invertebrate predator (*Ischnura elegans*). Purified MC-LR exerted a higher growth inhibitory effect on *S. quadricauda* compared to crude extract with the same concentration of MC-LR, while neither treatment affected its chlorophyll-a content or survival of *D. pulex*. Crude extract reduced grazing and survival of *D. pulex and I. elegans*, respectively. The combined effect of higher temperature and crude extract reduced *I. elegans* survival by 50%*.* Increased temperature reduced prey handing time in *I. elegans* by 49%, suggesting a higher predation rate. However, warming together with higher concentrations of crude extract jointly increased zooplankton grazing and reduced damselfly predation. Taken together, these results suggest crude extract, and not necessarily microcystin, can affect survival and productivity in freshwater species, although these effects may vary unevenly across trophic levels. Our findings highlight the importance of complex ecological mechanisms by which warming can exacerbate toxic effects of cyanobacterial bloom extracts on survival and functions among species in eutrophic freshwaters.

**Supplementary Information:**

The online version contains supplementary material available at 10.1007/s00442-022-05277-7.

## Introduction

Harmful cyanobacterial blooms are among the biggest threats to freshwater quality (Brooks et al. 2016), biodiversity and ecosystem functioning globally (Amorim and Moura 2021; Briland et al. 2020; Shahmohamadloo et al. 2020a). Freshwater cyanobacterial blooms have become more frequent and intense, producing microcystins (MCs) and other hazardous cyanotoxins that can cause serious public health, socio-economic and ecological problems in surface waters (Briland et al. 2020; Brooks et al. 2016). This scenario is expected to increase in many lakes due to the complex interactions between multiple stressors associated with anthropogenic climate change, eutrophication, and biological invasions (Schulhof et al. 2019; Sukenik et al. 2015; Urrutia‐Cordero et al. 2020), with UNDESA (2012) proposing an increase by at least 20% in the incidence of harmful algal blooms until 2050. For instance, synergistic interactions between human-induced nutrient enrichment, increased water temperature and thermal stratification have been associated with the intensification of microcystin-producing cyanobacterial blooms in freshwaters (Bui et al. 2018; Harke et al. 2016; Paerl 2008). Although efforts to understand the ecological consequences of microcystin-producing blooms on freshwater ecosystems have been considerable (Ibelings et al. 2008; Wang et al. 2017), one major unanswered question remains the ecological importance of cyanotoxins relative to other stressors in the effects observed during freshwater harmful blooms (Briland et al. 2020; Ibelings et al. 2008). Therefore, it is unclear how exposure to increased cyanotoxin concentrations may affect survival of freshwater species and the ecosystem processes in which they are involved.

MCs are the most prominent group of cyanotoxins produced by several bloom-forming cyanobacteria in freshwaters (Chorus and Welker 2021; de Figueiredo et al. 2004; Janssen 2019). They occur naturally either as cell-bound, intracellular toxins or as dissolved extracellular toxins in freshwater bodies (Pham and Utsumi 2018). The intracellular MC content in intact cyanobacterial cells is usually several orders of magnitude higher than dissolved levels in water bodies, where concentrations rarely exceed 10 µg/L (Chen et al. 2017; Zhu et al. 2021). MC occurrence in surface waters has been linked to severe adverse effects in humans and a wide range of animals, such as birds, amphibians, fish, and zooplankton (Chen et al. 2009; Jos et al. 2005). Therefore, the World Health Organisation (WHO) recently recommended provisional guideline values of 1 µg/L and 12 µg/L as lifetime and short-term permissible MC-LR concentrations in drinking, and 24 µg/L for recreational waters (Chorus and Welker 2021). A few reports suggest that occasional occurrence of dissolved MC concentrations in surface waters can reach > 1 mg/L in specific situations, such as during cell lysis of senescent blooms, or immediately after algicide treatment (Babica et al. 2007; Kotak and Zurawell 2007; Sivonen and Jones 1999). However, other studies have shown rapid degradation of dissolved MC concentrations, such as > 50% within 5 days (Zheng et al. 2004). This rapid degradation suggests that high concentrations of dissolved MCs are unlikely to persist for longer periods in freshwaters and so typical environmental concentrations of dissolved MCs rarely exceed the above-mentioned guideline values recommended for human exposure (Chen et al. 2017; Chorus and Welker 2021). However, it is unclear whether the guideline values for human exposure can serve to estimate risks to populations of aquatic organisms. Hence, it is important to understand the effects of low concentrations of dissolved microcystins on survival and functions among freshwater species.

Freshwater species mediate important ecological processes, such as primary production, herbivory, and predation in aquatic ecosystems (Galic et al. 2017). The dynamics of these processes have been associated with the overall productivity and functioning in aquatic systems (McKie et al. 2009; Oliver et al. 2015). However, evidence of the impact of dissolved MCs at sublethal concentrations on freshwater species is yet controversial and may potentially influence their survival and function (Burkholder et al. 2018; Chen et al. 2005; Shahmohamadloo et al. 2020b). Although several explanations have been proposed regarding the ecological role of MC production in competition (Babica and Maršalek 2006) and cyanobacteria–zooplankton interactions (Ger et al. 2016; Omidi et al. 2018), existing empirical evidence on these propositions is still currently limited and contradictory (Moustaka-Gouni and Sommer 2020). Previous studies have linked MC exposure with a variety of adverse effects in different freshwater taxa, including allelopathic inhibition of growth and photosynthetic pigments in algae (El-Sheekh et al. 2010), altered feeding and life history in zooplankton (Ghadouani et al. 2004; Smutná et al. 2014), and bioaccumulation and oxidative stress in molluscs (Zhang et al. 2016). These studies demonstrated the effects of either intact cells, crude extracts, or purified toxin on single species at concentrations well above those typically found in freshwaters (Burkholder et al. 2018). Therefore, findings from such studies may be difficult to extrapolate to the field as inferences drawn from single-species or single-stressor studies may be inadequate for generalising to more complex situations (Edwards and Pascoe 2018).

Climate change has been predicted to increase water temperature and thermal stratification for many lakes (Urrutia‐Cordero et al. 2020). Consequently, increased water temperature may act independently (Woodward et al. 2010) or in conjunction with other stressors, such as dissolved MC concentrations, to affect survival and key functions among aquatic species (Burkholder et al. 2018). Increased water temperature will accelerate rates of metabolic and cellular activities in freshwater organisms and such effects may vary across species depending on species-specific thermal thresholds (Brown et al. 2004). In combination with a second stressor, this change in metabolism may result in non-linear synergistic or antagonistic effects which may lead to unexpected ecological outcomes at the community level (Vinebrooke et al. 2004), potentially influencing trophic interactions (Woodward et al. 2010).

To investigate the potential impacts of warming and cyanotoxins on species survival and trophic interactions, we tested the combined effects of temperature and dissolved MC concentrations (both using purified MC-LR and crude extract of *Microcystis aeruginosa*) on survival and processes relating to resource–consumer interactions in three freshwater taxa: *Scenedesmus quadricauda* (green alga), *Daphnia pulex* (zooplankton grazer) and *Ischnura elegans* (predatory damselfly larva). These species occupy significant positions in the freshwater food webs, and have been widely used as sentinel experimental models in many ecotoxicological studies (Šulčius et al. 2017). We conducted a suite of factorial laboratory experiments, consisting of three temperatures (15 °C, 20 °C, 25 °C) and two MC treatments (purified MC-LR and crude extract) at environmentally realistic concentrations (0.01–10 µg/L). The temperature range tested is in accordance with those reported in previous studies, corresponding to characteristic average (15 °C), slightly elevated (20 °C), and elevated (25 °C) thermal conditions in European freshwaters (Henry et al. 2017). MC concentrations tested were in accordance with existing evidence showing that dissolved MCs typically occur at relatively low concentrations ranging from 10 s of ng/L to a few µg/L in water bodies (Chen et al. 2013; Wei et al. 2020). We monitored effects of both stressors on (i) primary productivity in the algal species by measuring algal growth inhibition and photosynthetic chlorophyll-a; (ii) survival and grazing rate in a daphnid species; and (iii) survival and predation rates in damselfly larva.

First, based on previous studies (Burkholder et al. 2018), we expected negative effects of sublethal MC concentrations on survival, primary production, grazing and predation in our study species. This is because existing studies have associated exposure to low concentrations of chemical stressors below recommended safe thresholds with non-linear patterns of negative effects on aquatic species (de Souza Machado et al. 2017; Rivetti et al. 2016). Therefore, we refer to our chosen concentrations as “low” relative to the experimental concentrations used in previous studies and relative to short-term environmental concentrations sometimes observed at the peak of bloom senescence (Chen et al. 2005; DeMott et al. 1991; Shahmohamadloo et al. 2020a). Second, we hypothesised that survival, grazing and predation would be reduced for the zooplankton and predator species at higher temperatures when thermal thresholds are exceeded (Heugens et al. 2003; Kenna et al. 2017; Verheyen et al. 2019). Finally, we predicted interactive effects of temperature and dissolved MCs on survival and feeding-related processes in our study species, such that negative effects of toxins are exacerbated at higher temperatures. This third hypothesis is based on increased metabolic rates at higher temperatures, leading to greater active uptake and metabolism of the toxin (Zurawell et al. 2005).

## Materials and methods

### Cyanobacterial culture

A toxigenic cyanobacterial strain, *Microcystis aeruginosa*, (CCAP/14/50/16) was obtained from the Culture Collection for Algae and Protozoa (CCAP), Scottish Marine Institute, Scotland. *M. aeruginosa* was cultivated in the laboratory as batch cultures under axenic conditions in 250 mL Erlenmeyer flasks containing 150 mL autoclaved BG-11 culture media prepared in line with the CCAP’s recipe. These cultures were maintained under constant temperature (25 ± 1 ºC), light intensity (25 µmol quanta m^−2^ s^−1^) and photoperiod cycle (12 h: 12 h light/dark) in a shaking incubator until cells were harvested. Before harvest, daily cell density was monitored by spectrophotometric measurement of the optical density (OD) at 680 nm as well as by light microscopy using a compound microscope and Sedgewick-Rafter Counting Chamber. The relationship between the daily spectrophotometric OD measurement and the cell density was established by fitted linear regression (*R*^2^ = 0.97). Cells were harvested at the stationary phase and cell density was estimated by spectrophotometry before the cells were lysed through a freeze–thaw cycle to release the intracellularly bound MC content (note that this may potentially release other unidentified water-soluble cellular components). The total MC content in the crude *Microcystis* extract was quantified and expressed as MC-LR using a semi-quantitative Adda-specific MC ELISA kit (CAT No. ALX-850–391-KI01, Enzo Life Sciences) according to Sarnelle et al. (2010). The purified MC-LR (CAS No. 101043–37-2, purity ≥ 95%) was supplied by Cayman Chemical Company, UK.

### Algal primary production and photosynthetic chlorophyll-a

*Scenedesmus quadricauda* (A950) was purchased from Sciento Scientific Ltd., Manchester, UK and maintained in BG-11 media on a cool white fluorescent lamp-illuminated shelf in a controlled temperature room kept at 21 ± 1 °C; 54 µmol quanta m^−2^ s^−1^ and 14 h:8 h light/dark photoperiod. Daily cell density increases were monitored by spectrophotometric measurement of the optical density (OD) at 680 nm and using a compound microscope and Sedgewick-Rafter Counting Chamber.

We set up 40 experimental replicates consisting of 100 mL cultures of *S. quadricauda* in 150 mL Erlenmeyer flasks for each temperature, in accordance with the OECD guidelines (OECD 2011). In all, a total of 120 replicates across three temperatures (15, 20 and 25 °C) was established. Each replicate culture was made up of sterile BG-11 medium, a known concentration of MC-LR (either in the crude *Microcystis* extract or the purified toxin) and 10 mL of the *S. quadricauda* inoculum (2.64 × 10^4^ cells/mL) which had been incubated and acclimated as an exponentially growing culture for 4 days before the experiment. We tested the effects of five concentrations of the purified MC-LR treatment (0.01, 0.1, 0.5, 1.0 and 10.0 µg/L) and the equivalent concentrations in crude *Microcystis* extracts on the growth inhibition and photosynthetic pigments content of *S. quadricauda* at three different temperatures: 15, 20 and 25 °C. Note that the crude *Microcystis* extract represents a more ecologically relevant mixture of secondary metabolites released during cell lysis including MC. Throughout the study, we express the crude extract concentration in terms of the concentration of MC that it contains. Each treatment concentration and control had four replicates. The control group consisted of a blank control and a solvent control. The blank control consisted of all the experimental conditions without MC treatment, while the solvent control also contained 0.1 mL of methanol (0.1%, *v*/*v*) to distinguish the effects of the organic solvent used as diluent from the effects of the purified toxin. The experiment was incubated at three temperatures (15, 20 and 25 °C) and constant light intensity (54 µmol quanta m^−2^ s^−1^) over a fixed photoperiod (14 h:8 h hour light/dark cycle) for 72 h. On each day of the experiment, 5 mL of the samples was taken and replaced with equal volume of the growth medium. The daily biomass increase for each replicate was estimated from the linear regression between the spectrophotometric OD_680_ data and the microscopic cell counts (Ma et al. 2015). The percentage growth inhibition of *S. quadricauda* was calculated from the specific growth rate of the treatment relative to the solvent control (Wang et al. 2017). The effects of temperature and MC treatments on chlorophyll-a content as a surrogate measure of photosynthetic processes were determined by taking the spectrophotometric optical density reading of the methanol extracted samples at OD_440_, OD_645_, OD_652_ and OD_663_. The pigment content was calculated as described by Fang et al. (2018).

### Zooplankton survival and grazing

Adults of the cladoceran zooplankton, *Daphnia pulex*, were obtained from Blades Biological Ltd., Kent, UK. Daphnids were cultured in a controlled temperature (CT) room at 21 ± 1 °C and 16 h:8 h light:dark photoperiod for 3 weeks, until third broods of neonates were produced by each adult female. Fifteen adult animals were maintained in 500 mL glass beakers filled with 300 mL of “Aachener Daphnien Medium” (ADaM), a modified artificial media for zooplankton (Klüttgen et al. 1994). Daphnids were fed with 10 mL of *S. quadricauda* (5.0 × 10^6^ cells per mL) three times weekly. The culture medium and algal food material were renewed every 3 days and the neonates produced were counted and removed daily (Rohrlack et al. 2001). To ensure standardised maternal conditions, only neonates produced at the third brood and after were used for experiments.

A 48-h static acute toxicity test was conducted to test the combined effects of temperature and MC treatments on the survival of *D. pulex* following the OECD guidelines (OECD 2004). Daphnid neonates less than 24 h old and taken from the female’s third brood were exposed to five treatment concentrations (0.01, 0.1, 0.5, 1.0, and 10.0 µg/L) of purified MC-LR and three concentrations of crude *Microcystis* extract (0.01, 0.1, and 0.5 µg/L). Tests were conducted in 100 mL plastic beakers, containing 50 mL of the experimental medium (ADaM), MC treatments and 10 daphnid neonates per cup. Daphnids were incubated under controlled conditions at three different temperatures (15, 20, and 25 °C), 54 µmol quanta m^−2^ s^−1^ and 14 h:10 h hour light/dark cycle. Six replicates of a control, consisting of the same experimental conditions without MC treatments, were kept at each temperature, while three replicates per MC concentration were tested at each temperature (42 replicates in total, see Table S1 for a schematic of the experimental designs). The proportion of immobilised neonates per replicate was counted as a surrogate measure of mortality (endpoint) at 24 and 48 h (Luo et al. 2018).

The grazing inhibition tests in *D. pulex* were conducted in accordance with the method described by Jesus et al. (2014). Five 4-day-old daphnids were exposed to the same concentrations of either MC-LR or crude *Microcystis* extract as those used for the survival experiments at the same three temperatures (15, 20, and 25 °C) in 50 mL plastic beakers. Each beaker contained a 20 mL solution made up of a combination of the test medium (ADaM), MC treatments, and 5 mL of *Scenedesmus* culture (1.45 × 10^6^ cells/mL) as a food source for daphnids. We used two experimental treatments without MC: the blank treatment experienced the same experimental conditions as the MC replicates but without daphnids or MC to measure algal growth in the absence of grazing and MC. The control treatment experienced the same experimental conditions as the MC replicates and included five daphnids but no MC treatments were added to measure grazing in the absence of MC. These experimental conditions were all made in four replicates across the control, the blank and each combination of MC concentration and temperature. To reduce the influence of light on algal growth during the experiment, the tests were incubated in the dark while animals were only allowed to graze for 24 h. At the end of the test, daphnids were gently removed with the aid of a plastic pipette, while individual beakers were shaken vigorously to ensure uniform concentration of algae before determining the cell density by taking the spectrophotometric optical density (OD_680_) at 680 nm. The individual grazing rate for each animal was estimated relative to the blank control and expressed as the change in the cell density during the 24 h grazing test (Allen et al. 1995).

## Damselfly survival and predation

Blue-tailed damselfly larvae, *Ischnura elegans*, were collected between June and July 2019 from ponds located at None-Go-By Farm (53° 52′ 33.75'' N, 1° 38′ 37.57'' W) in Horsforth, West Yorkshire, UK. Larvae were sampled using a pond net, sorted in a plastic tray, and transported to the laboratory in a small cool box containing pond water for further identification using taxonomic keys (Cham 2012). *Ischnura elegans* were acclimated to laboratory conditions in plastic tanks containing 100 mL of aged, dechlorinated tap water, maintained at constant temperature of 20 °C and 14 h:10 h light:dark photoperiod cycle. Larvae were fed ad libitum with *Daphnia magna*, while a wooden dowel rod was used to provide a perch for the larvae in each tank (Villalobos-Jiménez et al. 2017).

A 24 h functional response experiment was conducted to test the effects of MC and temperature on survival and prey consumption using methods in Villalobos-Jiménez et al. (2017). Larvae with a size range of 1.5–3.1 mm at the 9th–11th instars were acclimated to the experimental temperature and starved for 24 h before the start of the experiment. Animals were housed individually in 200 ml plastic containers in aged, dechlorinated tap water, incubated at 14 h:10 h light:dark photoperiod cycle, and exposed to two concentrations of crude extract treatments (0.05 and 0.2 µg/L) or a control containing no extract at two different temperatures (15 and 25 °C). Data were also collected at 20 °C, but due to a lack of size-matched individuals, those data were not analysed. Purified toxin was not available for this experiment. Individual larvae were fed with one of five different prey densities, consisting of 5, 10, 15, 30, and 50 *D. magna* individuals (body size 1.0–1.4 mm) as prey. Seven replicates of each prey density were used for the experiment at 15 °C and 25 °C. Each damselfly was only used in one replicate. At the end of the 24 h exposure, the numbers of *Daphnia* consumed were counted.

### Data analysis

Algal response data (growth inhibition and chlorophyll-a pigment) were checked for normality assumptions using the Shapiro–Wilk test. The 72 h percentage algal growth inhibition data were log-transformed to avoid non-normal distribution of the residuals and general linear models were fitted to test the effects of toxin and temperature on both response variables.

Survival of *D. p*ulex individuals exposed to MC treatments were found to be greater than 50%, therefore survival of daphnids could not be estimated as the median lethal concentrations (LC_50_); a concentration that would kill 50% of the exposed population. Instead, Cox proportional hazard (CPH) models in the *survival* (Therneau 2015; Therneau and Grambsch 2000) and *survminer* (Alboukadel et al. 2019) packages in R were fitted to test for variation in time to death across toxin and temperature treatments. The 24 h grazing in zooplankton was checked for normality assumptions and analysed by fitting general linear models. Significant differences among treatment and temperature levels were determined using Tukey’s multiple comparison test in the *multcomp* package in R (Hothorn et al. 2008).

The individual and combined effects of temperature and crude *Microcystis* extract treatments on the survival of *I. elegans* during the 24 h experiment were tested by fitting a binary logistic regression. We explored the differences in the effects of temperature and crude extract treatments on the attack rate and handling time of *I. elegans* using the *frair* package (Pritchard 2017; Pritchard et al. 2017) in R with a Benjamini–Hochberg correction to the resulting *p* values. Prey consumption rates followed a type II functional response. Since there was no prey replacement during the experiment, we estimated handling time (*h*) and attack rate (*a*) following Rogers’ type II formula (Rogers 1972) with Lambert’s W function (Bolker 2008). We tested the difference in the handling times and attack rates between treatments using bootstrapping.

## Results

### Algal growth inhibition and chlorophyll-a

**Fig. 1:**
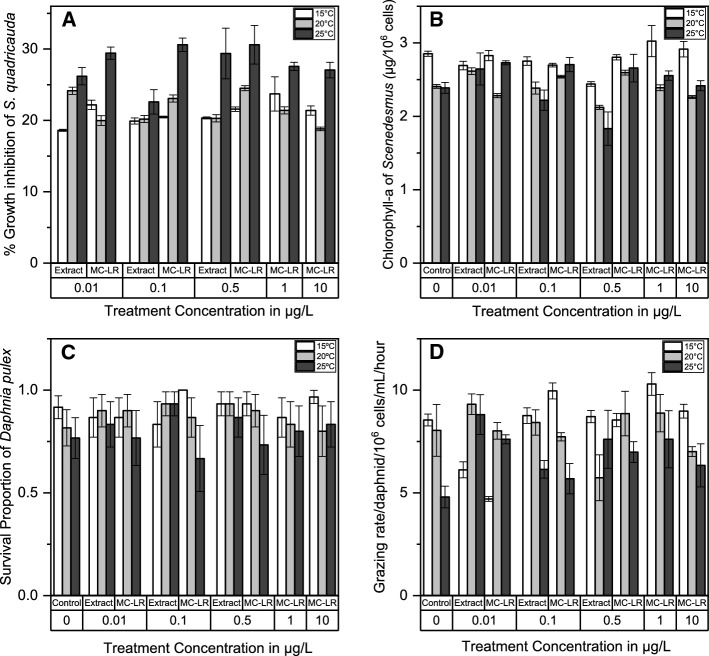
Effect of temperature and treatment concentration on **A** growth inhibition and **B** chlorophyll-a in the freshwater alga *Scenedesmus quadricauda* and survival (**C**) and grazing rate (**D**) in the zooplankton *Daphnia pulex*. Note that crude extracts contained the same concentration of MC-LR as the treatment with purified MC-LR and that each point represents mean ± SEM error bars in **A**, **B** and **D** but mean ± 95% binomial confidence levels error bars in **C**. For sample sizes in each experiment, please see Table S1

Both purified MC-LR and crude extract containing a corresponding amount of MC-LR inhibited the growth of *S. quadricauda* by 20–30% to a similar extent at all concentrations tested (Fig. 1A; Table 1). The effect of pure MC-LR was slightly but significantly more pronounced than that of crude extract containing the same concentration of MC-LR (*p* = 0.001; Fig. 1A; Table 1). Inhibition was significantly greater at 25 °C than at the two lower temperatures tested (*p* < 0.001; Fig. 1A).

**Table 1:** GLM output showing the parameter estimates with associated standard errors (SE), *t* values, and *p* values of the main and interactive effects of (MC-LR and crude extract corresponding to the same concentration of MC-LR) treatments and temperature on the percentage growth inhibition and chlorophyll-a content of *S. quadricauda* and grazing rate in *D. pulex*, respectively, relative to the controls

Species	Response	Parameter	Estimate	SE	*t* value	*p* value
*Scenedesmus quadricauda*	% Growth inhibition	Intercept	2.541	0.0657	38.67	** < 0.001**
		Purified MC_LR	0.094	0.0277	3.39	**0.001**
		Concentration	− 0.009	0.0042	− 2.18	**0.03**
		Temperature	0.028	0.0031	8.92	** < 0.001**
*Scenedesmus quadricauda*	Chlorophyll-a	Intercept	2.781	0.0798	38.84	** < 0.001**
		Extract	− 0.135	0.0833	− 1.62	0.109
		Purified MC_LR	0.110	0.0814	1.35	0.180
		Concentration	− 0.014	0.0091	− 1.52	0.131
		Temperature	− 0.032	0.0064	− 4.98	** < 0.001**
*Daphnia pulex*	Grazing rate	Intercept	14.616	2.639	3.42	** < 0.001**
		Extract	− 6.185	3.048	− 2.03	**0.045**
		Purified MC-LR	− 3.418	2.894	− 1.18	0.240
		Concentration	− 0.036	0.061	− 0.58	0.562
		Temperature	− 0.374	0.129	− 2.90	**0.005**
		Extract × temp	0.340	0.149	2.28	**0.025**
		Purified × temp	0.209	0.142	1.48	0.143

The *p* values in bold are significant, if *p* < 0.05

Our results showed no evidence of statistically significant effects of purified MC-LR (*p* = 0.180), crude *Microcystis* extract (*p* = 0.109) or toxin concentration (*p* = 0.131) on the chlorophyll-a pigment content of *S. quadricauda* (Fig. 1B; Table 1). However, increased water temperature from 15 °C to 25 °C significantly reduced chlorophyll-a pigment content of *S. quadricauda* (*p* < 0.001; Fig. 1B). The highest chlorophyll-a content in this study was observed at 15 °C (*p* < 0.001; Fig. 1B).

### Zooplankton survival and grazing

Neither purified MC-LR nor crude *Microcystis* extract treatments had significant effects on the survival of *D. pulex* at the range of concentrations tested in this study when compared with the control (Table 2, Fig. 1C). However, increased water temperature from 15 °C to 25 °C significantly reduced survival of *D. pulex* individuals (*p* < 0.001; Table 2, Fig. 1C). Crude *Microcystis* extract only (*p* = 0.045), but not the purified MC-LR (*p* = 0.280), significantly reduced the grazing rate of *D. pulex* relative to the control (Table 1, Fig. 1D). However, these effects did not differ significantly across the range of toxin concentrations tested in this study (*p* = 0.562; Table 1, Fig. 1D). Interestingly, the crude extract increased the grazing rate of *D. pulex* relative to the control (*p* = 0.025, Fig. 1D), albeit without a trend across treatment concentrations and temperature.

**Table 2:** Logistic regression models and their associated parameter estimates (odds ratios), *Z* value 95% confidence intervals (95% CI) and *p* values summarising the combined effects of two MC treatments (purified MC-LR and crude *Microcystis* extract containing the same concentration of MC-LR) and temperature on the survival of the microcrustacean zooplankton, *D. pulex* and damselfly larva, *Ischnura elegans*

Species	Predictors	Odds ratios	*Z* value	95% CI	*p* value
*Daphnia pulex*	Intercept	0.03	− 6.57	0.01–0.08	**< 0.001**
	Extract	0.60	− 1.81	0.34–1.04	0.070
	Purified	0.95	− 0.21	0.58–1.57	0.835
	Concentration	0.98	− 0.61	0.91–1.05	0.539
	Temperature	1.10	4.04	1.05–1.16	**< 0.001**
*Ischnura elegans*	Intercept	61.53	2.14	1.82–4450	**0.032**
	Extract	360.16	2.10	1.42–113,277	**0.036**
	Temperature	0.94	− 0.66	0.78–1.13	0.512
	Extract × temp	0.71	− 2.71	0.55–0.91	**0.007**

Bold *p* values indicate *p* < 0.05

### Damselfly survival and predation

**Fig. 2:**
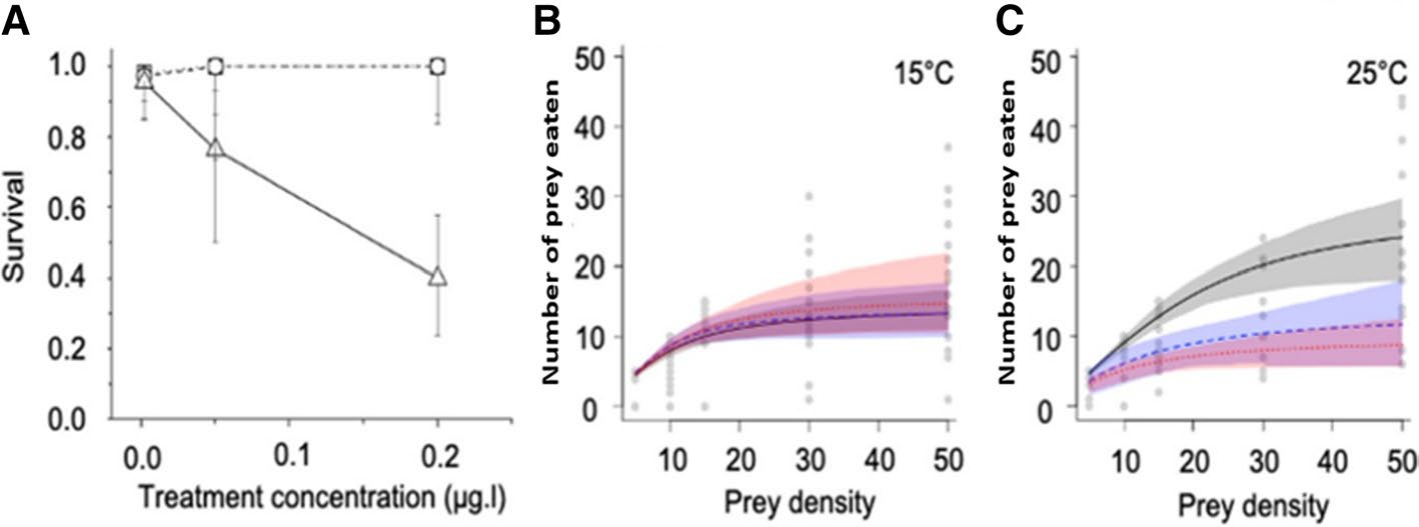
Combined effects of temperature and crude extract on survival and predatory functional response of damselfly larvae, *I. elegans*. **A** Survival (circles = 15 °C and triangles = 25 °C) and error bars represent 95% confidence levels. **B** Predation at 15 °C (grey = 0 µg/L, blue = 0.05, red = 0.2 µg/L), with considerable overlap. **C** Predation at 25 °C (grey = 0 µg/L, blue = 0.05, red = 0.2 µg/L), where the grey control has a higher predation rate than the 0.05 and 0.2 µg/L treatments. In **B** and **C**, the shaded areas represent 95% confidence levels. **B** and **C** are functional response plots, showing the number of prey consumed when those prey are presented to a predator at a range of densities. If a predator was able to consume all prey regardless of density, there would be a 1:1 relationship while deviation from the 1:1 relationship indicates a limiting effect of attack rate or handling time. For sample sizes in each experiment, please see Table S1

The crude *Microcystis* extract treatment (from 0.05 µg/L to 0.2 µg/L) significantly reduced survival of *I. elegans* relative to the control, albeit only at 25 °C and not at 15 °C (*p* = 0.036; Fig. 2A, Table 2). The observed effect was far more pronounced in the crude extract treatment containing the higher concentration of MC-LR. Overall, the survival odds ratio of *I. elegans* in the control was approximately 6 times higher than in presence of the extract (Table 2). Although increased temperature alone had no significant overall effect on the survival of *I. elegans* (*p* = 0.512; Fig. 2A, Table 2), there was a significant interaction between increased temperature and crude extract, such that the highest extract concentration containing 0.2 µg/L MC-LR and increased temperature at 25 °C jointly reduced survival of *I. elegans* by almost 50% compared to the control (*p* = 0.007; Fig. 2A, Table 2).

Figure 2B, C shows the functional response curves resulting from experiments at 15 °C and 25 °C, respectively, under control (0 µg/L), 0.05 µg/L and 0.2 µg/L of crude extract. In each plot, a curve closer to the 1:1 line indicates higher rates of predation (predator consumes a greater proportion of available prey in the time period) that may correspond to higher attack rates, lower handling times, or both. In the absence of crude extract, there was no difference in the attack rate of *I. elegans* between the two temperatures (*p* = 0.92; Table 3). However, the prey handling time of *I. elegans* was significantly reduced by 49% when temperature increased to 25 °C, indicating a higher predation rate (*p* < 0.001; Table 3; note the higher asymptote on the grey control line in Fig. 2C compared to Fig. 2B). When looking only at the 15 °C experimental treatments, functional response curves showed no significant effects of increased crude extract concentrations compared to the control on the attack rate (control vs 0.05 µg/L: *p* = 0.67; control vs 0.2 µg/L: *p* = 0.45) or prey handling time of *I. elegans* (control vs 0.05 µg/L: *p* = 0.64; control vs 0.2 µg/L: *p* = 0.92; Table 3; note the overlapping confidence intervals in Fig. 2B). At 25 °C, a crude extract concentration corresponding to 0.2 µg/L MC-LR significantly increased the prey handling time compared to the control at 25 °C (*p* < 0.001; Table 3; note the higher predation rate indicated by the grey control curve compared to the red 0.2 µg/L curve in Fig. 2C)*.* No significant effect was observed on the attack rate of *I. elegans* under increased crude extract concentration at 25 °C (*p* = 0.38; Table 3).

**Table 3:** Pairwise comparison of attack rate and handling time coefficients obtained from the functional response of *I*. *elegans*, showing estimate, *Z* values with the associated standard error and Benjamini–Hochberg correction adjusted *p* values at 0.05 µg/L, 0.2 µg/L with control and two temperatures 15 and 25 °C

Pairwise comparison	Attack rate				Handling time			
	*Estimate*	*SE*	*Z* value	*p* value	Estimate	SE	*Z* value	*p* value
15 °C (control): 15 °C (0.05)	− 0.470	0.701	− 0.671	0.656	0.010	0.014	0.729	0.635
15 °C (control): 15 °C (0.2)	− 0.104	0.944	− 1.105	0.448	− 0.004	0.015	− 0.236	0.917
15 °C (control): 25 °C (control)	0.085	0.598	0.143	0.916	0.058	0.013	4.526	**<** 0.001*
15 °C (control): 25 °C (0.05)	1.116	0.633	1.761	0.180	− 0.006	0.030	− 0.188	0.917
15 °C (control): 25 °C (0.2)	1.228	0.658	1.857	0.158	− 0.052	0.039	− 1.326	0.370
15 °C (0.05): 15 °C (0.2)	− 0.574	0.960	− 0.598	0.687	− 0.014	0.013	− 1.034	0.475
15 °C (0.05): 25 °C (control)	0.550	0.621	0.886	0.537	0.048	0.010	4.597	**<** 0.001*
15 °C (0.05): 25 °C (0.05)	1.580	0.661	2.392	0.067	− 0.016	0.030	− 0.540	0.706
15 °C (0.05): 25 °C (0.2)	1.693	0.682	2.471	0.067	− 0.062	0.038	− 1.617	0.226
15 °C (0.2): 25 °C (control)	1.129	0.887	1.272	0.375	0.062	0.012	5.084	**<** 0.001*
15 °C (0.2): 25 °C (0.05)	2.159	0.116	2.368	0.067	− 0.002	0.030	− 0.071	0.943
15 °C (0.2): 25 °C (0.2)	2.267	0.928	2.441	0.067	− 0.048	0.039	− 1.239	0.375
25 °C (control): 25 °C (0.05)	1.031	0.544	1.891	0.158	− 0.064	0.029	− 2.191	0.097
25 °C (control): 25 °C (0.2)	1.139	0.573	1.986	0.141	− 0.109	0.038	− 2.904	** <** 0.05*
25 °C (0.05): 25 °C (0.2)	0.099	0.610	0.164	0.917	− 0.046	0.047	− 0.985	0.487

Bold *p* values indicate *p* < 0.05

## Discussion

This study tested the combined effects of increased environmental temperature and dissolved MC concentrations in purified MC-LR and crude extract treatments on survival, productivity, and resource–consumer interactions in three important freshwater species covering three trophic levels. Our results showed increased crude extract concentrations and elevated temperature jointly reduced the survival of *I.* elegans, while the predation rate of *I. elegans* increased only at higher temperatures. Increased temperature in combination with increased crude extract concentrations reduced predation rate in *I. elegans*, but increased grazing rate in *D. pulex*. Independently, increased temperature inhibited algal growth and photosynthetic chlorophyll-a pigment in *S. quadricauda* and reduced survival of *D. pulex*. However, purified MC-LR demonstrated a slight, but statistically significant higher inhibitory effect on the growth of *S. quadricauda* compared to crude extract with corresponding MC-LR concentrations. Taken together, our results suggest that the observed negative effects of our crude extract treatment in this study may not be entirely due to the dissolved MC concentrations alone. The potential effects of other unidentified secondary metabolites and their combined effects with one another and temperature cannot be ruled out.

### Effect of low environmental MC-LR concentrations

We observed a significant inhibitory effect of purified MC-LR on the growth of *S. quadricauda* while the photosynthetic chlorophyll-a content was unaffected by either crude extract or purified MC-LR treatments. This high inhibitory effect of the purified MC-LR on the growth of *S. quadricauda* is accordance with previous findings in the literature (Babica et al. 2007; El-Sheekh et al. 2010; Pflugmacher 2002) and supports the argument that MC may potentially be involved in the allelopathic interactions between cyanobacteria and other phytoplankton during interspecific competition (Omidi et al. 2018). Several studies have proposed that MC may act as a potential allelochemical (Babica and Maršalek 2006; Pflugmacher 2002), and Figueredo et al. (2007) have suggested that the toxin may be capable of shaping phytoplankton succession and community structure during freshwater blooms. The putative inhibitory role of MCs in allelopathic interactions has been attributed to its potential to inhibit growth, photosynthesis, and induce cellular oxidative stress during interspecific competition (Bittencourt-Oliveira et al. 2015; Omidi et al. 2018). However, the complex mechanisms behind these adverse effects are yet to be fully understood as evidence from previous laboratory studies indicate contrasting effects across MC treatments (Babica and Maršalek 2006; Ma et al. 2015).

Dissolved MC concentrations in purified toxins or crude extracts have been associated with a variety of adverse effects in zooplankton and aquatic invertebrates (Bownik 2016; Burkholder et al. 2018; Ger et al. 2016). However, in this study, low concentrations of the purified MC-LR had no effect on survival and grazing rate of *D. pulex*, but crude extract containing corresponding MC-LR concentrations exerted significant negative effects. This finding is in accordance with the observations of Jungmann (1994), suggesting that the negative effects observed in the crude extract were possibly not due to dissolved MC-LR concentrations but potentially other unidentified metabolites. The observed tolerance of *D. pulex* to purified MC-LR concentrations is in agreement with previous studies (Dao et al. 2018, 2010; Lürling and van der Grinten 2003), that reported little or no adverse effects of the purified toxin on survival of *Daphnia* at ecologically relevant concentrations. Lürling and van der Grinten (2003) and Chen et al. (2005) found no significant effects on survival of *D. magna* after a 21-day exposure to dissolved concentrations as low as 3.5 µg/L and 10 µg/L of the purified MC-LR, respectively. However, a longer term exposure to 5 µg/L and 50 µg/L of the purified MC-LR in the laboratory resulted in 10 and 60% reductions in survival of *Daphnia*, respectively, after 60 days (Dao et al. 2010). The range of the purified MC-LR concentrations (1–10 µg/L) tested in this study is several orders of magnitude lower than the lethal concentrations (LC_50_) reported for the purified toxin in *Daphnia* (Bui et al. 2020; DeMott et al. 1991). Moreover, there is increasing evidence that dissolved MC concentrations that persist in surface waters are usually < 10 µg/L (Graham et al. 2020; Loftin et al. 2016; Lürling and van der Grinten 2003; Skafi et al. 2021). Lürling and van der Grinten (2003) reported a range of 0.2 µg/L to 4.7 µg/L, which may persist for ~ 10 weeks depending on the predominant variant (Lahti et al. 1997). Although dissolved MC concentrations may be higher and tend to persist longer in tropical waters, where harmful cyanobacterial blooms dominate for longer period due to warmer temperature and eutrophication (Mowe et al. 2015). Hence, our results suggest low concentrations of the dissolved MCs are unlikely to cause acute toxicity in aquatic organisms, with a potentially greater role for other secondary metabolites released during cell lysis. Nevertheless, prolonged exposure in the environment may exert subtle chronic effects on individual survival, physiological fitness and key processes (Dao et al. 2018, 2010), which may propagate to higher levels of biological organisation (Nilsen et al. 2019).

### Effect of crude extract containing low concentrations of MC-LR

Growth inhibitory effect of crude *Microcystis* extract on *S. quadricauda* in this study was relatively low compared to the effect of purified MC-LR treatments, even though the concentration of MC-LR in the crude extract was the same as in the purified MC-LR treatments. This may be due to the nature of the crude extract and possibly attributed to the fact that the effects of MC-LR might have been masked by other unidentified co-occurring metabolites present in the crude extract (Janssen 2019). Crude cyanobacterial extracts have been shown to consist of a variety of heterogeneous metabolites produced by cyanobacteria other than MCs (Janssen 2019). The combined effects (be they additive, synergistic, or antagonistic) of these heterogeneous metabolites can mask or modulate potential adverse effects on aquatic organisms (Ibelings et al. 2008; Janssen 2019). However, contrary to our expectation, neither treatment affected chlorophyll-a content in our study. The quantification of chlorophyll-a and other photosynthetic pigments represents an important biomarker of photosynthetic and respiratory rates in algae and have been widely used to evaluate sensitivity of algae to xenobiotic exposures (Fang et al. 2018; Li et al. 2005). As reported in previous studies (Cheng et al. 2015; Wei et al. 2010), photosynthetic pigmentation is expected to serve as an early warning and a more sensitive signal to MC stress compared to growth inhibition. This is because changes in photosynthetic pigments are believed to occur earlier at the molecular level of the cells than growth inhibition during exposure to chemical stressors (Fang et al. 2018). However, in contrast to this expectation, our results indicate that low environmental MC concentrations in the range of those tested in this study are unlikely to affect the ability of algae to synthesise chlorophyll-a pigments during blooms. This observation could be due to the short duration of exposure in the present study, and we cannot rule out the fact that adverse effects on photosynthetic pigments might yet be observed as a consequence of a longer period of exposure.

In addition, the crude *Microcystis* extract treatments used in this study showed varied effects on survival and feeding in *D*. *pulex* and *I. elegans*. The observed variations in species’ sensitivity to concentrations of crude extract among these experimental models may be explained by differences in assimilation, metabolism and detoxification mechanisms in these organisms (Kozlowsky-Suzuki et al. 2012).

### Effects of increased environmental temperatures

Increased environmental temperature was associated with reduced survival and productivity in our model organisms. In support of the second hypothesis in this study, increased temperature reduced growth and photosynthetic chlorophyll-a content of *S. quadricauda*. The effect of increasing temperature was non-linear and followed a U-shaped dose–response pattern in the purified MC-LR treatments, possibly suggesting evidence of temperature-mediated hormesis. Hormesis has become an increasingly reported biphasic evolutionary phenomenon in many organisms (Erofeeva 2022), where low-dose exposure to environmental stress induces stimulation and high-dose inhibition, due to increased resilience or overcompensation (Agathokleous et al. 2019; Calabrese 2008; Forbes 2000). Increasing evidence has shown hormesis as an important adaptive biological response to a wide range of abiotic (including, temperature and light) and biotic natural stressors (cyanotoxins and allelochemicals) as well as anthropogenic pollutants in green plants and algae (Agathokleous 2021; Agathokleous et al. 2019; Erofeeva 2022). Here, our data consistently showed a U-shaped, non-linear effect across the range of low purified MC-LR concentrations tested, suggesting that increased temperature may have brought about a hormetic response of chlorophyll-a pigment to low concentrations of the purified MC-LR. These results are consistent with previous findings on temperature-mediated hormesis in plants (Agathokleous 2021; Agathokleous et al. 2019; Erofeeva 2022) and the effects of environmental warming on algae (Chalifour et al. 2014; Chalifour and Juneau 2011; Gomes and Juneau 2017; Larras et al. 2013). While warming beyond optimum thermal thresholds can induce adverse physiological changes at molecular (pigment alterations) and cellular levels (growth inhibition) in algae (Gomes and Juneau 2017), such effects are unlikely to be present in our study as our highest temperature (25 °C) is a standard culturing temperature and reductions in population growth rate only occur > 32 °C (Zargar et al. 2006). As a result, apparent negative effects of temperature are more likely due to interactions with cyanobacterial products.

Increased temperature had no effect on survival but was associated with reduced prey handling time of *I. elegans*, thereby increasing its predation rate. The observed thermal tolerance at 25 °C among *I. elegans* larvae could be due to the short exposure duration (24 h) in our experiment. Arguably, the exposure conditions and duration used in this study were possibly within the thermal preference for this freshwater predator. This finding is consistent with previous studies showing increased survival rate (Carbonell and Stoks 2020; Dinh et al., 2013) and thermal tolerance, CTmax (de Beeck et al. 2017), in *I. elegans* larvae exposed to a range of thermal conditions (20–24 °C) for more than 6 days in the laboratory. However, while 25 °C is considered an elevated thermal condition in the present study, our data suggests that this temperature is unlikely to be stressful for *I. elegans*. Carbonell and Stoks (2020) found a significant reduction in the survival of *I. elegans* exposed to 28 °C and above for 10 days, suggesting a longer term exposure to higher temperatures may be associated with significant adverse effects on survival of *I. elegans*. Moreover, our findings on increased predation rate in *I. elegans* at high temperature in this study are consistent with earlier studies (Thompson 1978; Wang et al. 2020), and may presumably be due to increased metabolic rate at higher temperature (Brown et al. 2004). Elevated temperature has been shown to accelerate the rate of metabolic processes, energy requirement and food intake in ectotherms (Brown et al. 2004; Galic et al. 2017; Stoks et al. 2014). Hence, higher predation rates observed in *I. elegans* at higher temperatures in this study may be associated with potential beneficial effects, including increased growth rate and immune response of the damselfly predator (Van Dievel et al. 2017; Wang et al. 2020).

We also observed reduced survival and grazing rates in *D. pulex* at higher temperatures. Our data in this study corroborate earlier findings by Müller et al. (2018), who demonstrated reduced survival among *D. magna* populations at temperatures above 29 °C, and reduced filtration rate in *Daphnia* when the optimal temperature (20 °C) was exceeded. Hence, these results suggest that the abundance of these keystone species may potentially be at risk as the climate becomes warmer. More importantly, vital processes that underpin ecosystem functions among key freshwater species may become impaired unless other more thermotolerant filter-feeders fill the gap as an expected ecosystem response (Ger et al. 2016).

### Combined effects of environmental temperature and crude extract concentrations

More ecologically significant and environmentally realistic findings than individual effects of temperature and toxins are those relating to their combined effects on freshwater species. Here, the interaction between the crude extract and increased temperature was associated with reduced survival of *I. elegans*. Higher temperatures lead to increases in metabolic activity and accelerate the toxicokinetics of chemical stressors in ectotherms (Brown et al. 2004; Noyes et al. 2009). Increased metabolic activity at higher temperatures presumably elevated the rate of uptake, assimilation, and toxicity of the bioactive compounds in the crude extract (Buchwalter et al. 2003; Kim et al. 2014; Kozlowsky-Suzuki et al. 2012). Such temperature-mediated toxicity could be responsible for the 50% decline observed in the survival of *I. elegans* exposed to high concentration of the crude extract at 25 °C in our study. Here, our finding supports the hypothesis on the combined effects of temperature and crude extract and corroborates earlier studies that demonstrated their combined effects on freshwater species (Kim et al. 2014; Lamb et al. 2019; Xiang et al. 2017). Hence, the present study suggests that warming may co-occur with a much broad range of cyanobacterial metabolites following bloom senescence leading to impaired survival and feeding among important species (Paerl and Huisman 2008; Walls et al. 2018).

We find evidence for antagonistic interactions between the effects of crude extract concentrations and increased temperature on the grazing rate of *D. pulex* and the predation rate of *I. elegans*. While increased temperature alone reduced prey handling time in *I. elegans*, the two stressors in combination jointly increased the prey handling time to a greater extent. Similarly, the combined effects of crude extract and elevated temperature further increased the grazing rates of *D. pulex*. Earlier studies have shown synergistic interactions between MCs and other environmental stressors may occur at lower dissolved MC concentrations, while antagonistic interactions were mostly observed at higher dissolved MC concentrations in other freshwater species (Liang et al. 2017, 2018; Wei et al. 2020). However, as dissolved MCs rarely occur in high concentrations under natural conditions (see above), a more realistic scenario for toxic blooms in freshwater bodies will be a combination of low concentrations of cyanobacterial metabolites with increased environmental warming.

## Conclusion

Here, we demonstrate using a suite of laboratory microcosms that increased temperature and environmentally relevant exposure to purified MC-LR, but more so to crude cyanobacterial extract containing MCs as well as other unidentified compounds, can affect survival and feeding in key freshwater species. We showed that low environmental MC concentrations adjudged to be safe for human health by the WHO can have species-specific effects for survival and ecosystem functions in *S. quadricauda* and possibly, as component of crude extract, in further freshwater taxa. Importantly, a more ecologically relevant effect on freshwater communities may become apparent when exposure to low concentrations of complex mixtures of cyanobacterial metabolites in the environment coincides with increased water temperature in eutrophic waters. Our results build on existing knowledge that suggests that other yet unidentified bioactive compounds present in cyanobacterial cells and not necessarily MC-LR may be responsible for negative effects observed during blooms. Hence, there is need for future studies to explore the potential toxic effects of bioactive compounds other than MCs in cyanobacterial crude extract.

## Supplementary Information

Below is the link to the electronic supplementary material.

Supplementary file1 (DOCX 17 KB)

## Data Availability

Raw data are available in the electronic supplementary information to the paper. Sources of animals and chemicals are provided in the text.

## Code availability

R code is available from the authors on request.
