# Supplementary material for: Combined effects of increased water temperature and cyanobacterial compounds exert heterogeneous effects on survival and ecological processes in key freshwater species
Source: Oecologia. 2022 Nov 7;200(3-4):515–28. doi: 10.1007/s00442-022-05277-7 (PMC9675649; doi:10.1007/s00442-022-05277-7)
Supplement: Supplementary file 1 — Supplementary file1 (DOCX 17 KB) [file 442_2022_5277_MOESM1_ESM.docx]

**[Electronic Supplementary Information]**

**Combined effects of increased water temperature and cyanobacterial compounds exert heterogeneous effects on survival and ecological processes in key freshwater species**

Oloyede A. Adekolurejo^1^, Matthew Floyd^1^, Alison M. Dunn^1^, Paul Kay^2^, Andrew P. Dean^3^, Christopher Hassall^1^

^1^School of Biology, Faculty of Biological Sciences, University of Leeds, LS2 9JT, UK.

^2^School of Geography, Faculty of Environment, University of Leeds, LS2 9JT, UK.

^3^Department of Natural Sciences, Faculty of Science and Engineering, Manchester Metropolitan University, M1 5GD

Corresponding author: [c.hassall@leeds.ac.uk](mailto:c.hassall@leeds.ac.uk) (CH)

**Supplementary Information**

**Table S1**: A summary of experimental design for all the three study species. For *Scenedesmus quadricauda*, the numbers refer to the number of replicate cultures. For *Daphnia pulex*, the numbers refer to the number of experimental pots and the numbers in parentheses refer to the number of animals per pot. For *Ischnura elegans*, the numbers refer to the number of animals used individually as replicates.

| Treatment |  | | Purified MC-LR | | | | Crude extract (MC-LR) | | |
| --- | --- | --- | --- | --- | --- | --- | --- | --- | --- |
| Species | Temperature (°C) | Control | 0.01 | 0.1 | 1 | 10 | 0.01 | 0.1 | 1 (µg/L) |
| *Scenedesmus quadricauda* | 15 | 4 | 4 | 4 | 4 | 4 | 4 | 4 | 4 |
|  | 20 | 4 | 4 | 4 | 4 | 4 | 4 | 4 | 4 |
|  | 25 | 4 | 4 | 4 | 4 | 4 | 4 | 4 | 4 |
|  |  |  |  |  |  |  |  |  |  |
| *Daphnia pulex* (survival) | 15 | 6 (10) | 3 (10) | 3 (10) | 3 (10) | 3 (10) | 3 (10) | 3 (10) | 3 (10) |
|  | 20 | 6 (10) | 3 (10) | 3 (10) | 3 (10) | 3 (10) | 3 (10) | 3 (10) | 3 (10) |
|  | 25 | 6 (10) | 3 (10) | 3 (10) | 3 (10) | 3 (10) | 3 (10) | 3 (10) | 3 (10) |
| *Daphnia pulex* (grazing) | 15 | 4 (5) | 4 (5) | 4 (5) | 4 (5) | 4 (5) | 4 (5) | 4 (5) | 4 (5) |
|  | 20 | 4 (5) | 4 (5) | 4 (5) | 4 (5) | 4 (5) | 4 (5) | 4 (5) | 4 (5) |
|  | 25 | 4 (5) | 4 (5) | 4 (5) | 4 (5) | 4 (5) | 4 (5) | 4 (5) | 4 (5) |
|  |  |  |  |  |  |  |  |  |  |
|  |  |  |  |  |  |  | Control | 0.05 | 0.2 (µg/L) |
| *Ischnura elegans* | 15 |  |  |  |  |  | 7 | 7 | 7 |
|  | 20 |  |  |  |  |  | 5 | 5 | 5 |
|  | 25 |  |  |  |  |  | 7 | 7 | 7 |
